# FRNet V2: A Lightweight Full-Resolution Convolutional Neural Network for OCTA Vessel Segmentation

**DOI:** 10.3390/biomimetics10040207

**Published:** 2025-03-27

**Authors:** Dongxu Gao, Liang Wang, Youtong Fang, Du Jiang, Yalin Zheng

**Affiliations:** 1 College of Electrical Engineering, Zhejiang University, Hangzhou 310027, China; youtong@zju.edu.cn; 2 School of Computing, University of Portsmouth, Portsmouth PO1 2UP, UK; 3 School of Automation and Electrical Engineering, Shenyang Ligong University, Shenyang 110180, China; wangliang@stu.sylu.edu.cn; 4 Key Laboratory of Metallurgical Equipment and Control Technology of Ministry of Education, Wuhan University of Science and Technology, Wuhan 430081, China; jiangdu@wust.edu.cn; 5 Department of Eye and Vision Science, University of Liverpool, Liverpool L69 3BX, UK

**Keywords:** optical coherence tomography angiography, blood vessel segmentation, neural networks, ConvNeXt V2, dataset

## Abstract

Optical coherence tomography angiography (OCTA) is an advanced non-invasive imaging technique that can generate three-dimensional images of retinal and choroidal vessels. It is of great value in the diagnosis and monitoring of a variety of ophthalmic diseases. However, most existing methods for blood vessel segmentation in OCTA images rely on an encoder–decoder architecture. This architecture typically involves a large number of parameters and leads to slower inference speeds. To address these challenges and improve segmentation efficiency, this paper proposes a lightweight full-resolution convolutional neural network named FRNet V2 for blood vessel segmentation in OCTA images. FRNet V2 combines the ConvNeXt V2 architecture with deep separable convolution and introduces a recursive mechanism. This mechanism enhances feature representation while reducing the amount of model parameters and computational complexity. In addition, we design a lightweight hybrid adaptive attention mechanism (DWAM) that further improves the segmentation accuracy of the model through the combination of channel self-attention blocks and spatial self-attention blocks. The experimental results show that on two well-known retinal image datasets (OCTA-500 and ROSSA), FRNet V2 can achieve Dice coefficients and accuracy comparable to other methods while reducing the number of parameters by more than 90%. In conclusion, FRNet V2 provides an efficient and lightweight solution for fast and accurate OCTA image blood vessel segmentation in resource-constrained environments, offering strong support for clinical applications.

## 1. Introduction

As the key physiological tissue of the eye, the health of the retina is closely related to the health of the eye. Optical coherence tomography angiography (OCTA) is an advanced non-invasive imaging technology [1]. According to the principle of OCT, it can generate three-dimensional images of retinal and choroidal blood vessels by analyzing light scattered by blood without injection of contrast agent. It is safe, convenient, and provides high-resolution vascular structure images. It is of great significance for the diagnosis and monitoring of ophthalmic diseases such as diabetic retinopathy, macular degeneration, and glaucoma.

**Figure 1:**
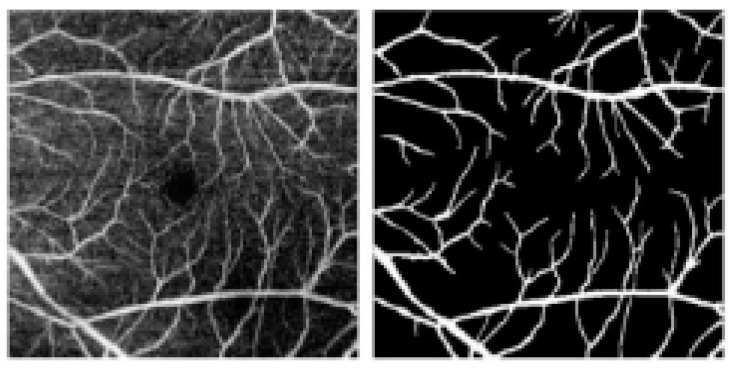
OCTA image and its segmentation map.

The original retinal OCTA images make it hard to clearly show the vascular structure. Professionals usually have to manually segment the vascular area, a process that is time-consuming and prone to subjective judgment [2]. Retinal blood vessels are complex, tree-shaped, small, fragile, and closely connected, with image noise further complicating segmentation [3]. Automatic retinal OCTA image segmentation offers a potential solution. It can lighten ophthalmologists’ workloads and enhance objectivity and consistency. This segmentation, either automatic or semi-automatic, differentiates retinal layers and vascular structures in acquired images, crucial for disease analysis and quantification. Figure 1 show an OCTA image and its segmentation map.

With the advancement of algorithms and technologies, more and more advanced algorithms are being applied to OCTA image segmentation. For example, convolutional neural network (CNN) [4] and full convolutional neural network (FCN) [5] are utilized, which has made the segmentation effect of OCTA images increasingly better. Subsequently, an encoder–decoder-based segmentation network is further proposed. This network achieves pixel segmentation by downsampling the input feature map to a lower resolution. Among encoder–decoder models, the U-Net model [6] is the most well-known segmentation network. U-Net has unique encoding and decoding layers connected by skip connections, integrating high-level and low-level features of images. This integration improves model accuracy and addresses the problem of vanishing gradients. Currently, many variants of the U-Net architecture have been proposed, such as U-Net++ [7], Attention U-Net [8], Dual Attention Network (DA-Net) [9], and Res-UNet [10], among others.

These above-mentioned algorithms are all based on deep-learning methods. Compared with traditional methods (threshold-based methods [11,12], clustering-based methods [13,14], and model-based methods [15,16]), they reduce the dependence on prior medical knowledge while significantly enhancing segmentation accuracy. However, all the deep-learning algorithm models mentioned above are based on the encoder–decoder architecture. The image is first downsampled and then upsampled. In each downsampling process, the number of channels in the convolutional layer usually doubles, resulting in a large number of parameters. These algorithms overlook the lightweight and speed requirements of the model. In scenarios such as emergency diagnosis, especially in remote areas with limited computing resources, the urgent need for real-time analysis emphasizes the necessity of fast and lightweight models.

Recently, the emergence of Transformers [17] has revolutionized the field of natural language processing. Thanks to its remarkable performance in this field, it has been applied to computer vision tasks, such as image segmentation, including Swin Transformer [18], TSNet [19], and SGAT-Net [20]. Owing to its ability to capture remote dependencies and contexts, Transformers have achieved remarkable results in segmentation tasks, with their accuracy and efficiency often surpassing those of traditional convolutional networks.

However, Liu et al.’s work, ConvNeXt [21], shows that well-designed convolutional networks can still achieve superior performance compared to Transformers. Examples include FARGO [22] and ASU-CNN [23]. ConvNeXt introduces a series of architectural improvements that enhance the capabilities of convolutional networks, enabling them to be competitive with Transformer-based models in a variety of tasks. On this basis, Sanghyun et al. proposed ConvNeXt V2 [24], which integrates a complete convolutional mask autoencoder framework and a new global response normalization (GRN) layer. The GRN layer is designed to normalize the global response of different feature maps. This helps to stabilize the training process and improve the model’s robustness. These enhancements significantly improve the performance of ConvNeXt on various recognition benchmarks, demonstrating that convolutional networks can outperform pure Transformer models in many scenarios when properly designed. In conclusion, while Transformers have made significant advances in image segmentation, recent advances in convolutional network architectures, such as ConvNeXt, ConvNeXt V2, and ASU-CNN, show that convolutional networks are still a robust and viable option.

To handle large parameters in encoder–decoder models and meet the demand for lightweight, fast models in areas with limited resources, we draw inspiration from recent research and propose FRNet V2. This uses ConvNeXt V2 and deep separable convolutions to build a network without upsampling or downsampling. Deep separable convolutions cut parameters and complexity in segmentation without weakening feature extraction, making the model lightweight. Additionally, a recursive mechanism is added to further reduce parameters and enrich feature representation, improving segmentation accuracy and the model’s lightweight feature.

This paper makes significant contributions in the following aspects:

- We design a new lightweight network for OCTA image segmentation, eschewing any upsampling–downsampling process. Employing the ConvNeXt V2 module as the core, along with deep separable convolutions and a recursive mechanism, this network bolsters the model’s feature extraction ability, slashes the number of parameters, and accelerates the model’s segmentation speed.
- We designed DWAM, a lightweight hybrid adaptive attention mechanism. It is divided into channel and spatial self-attention blocks. By introducing deep separable convolutions and recursive mechanisms, the mechanism becomes lighter, with faster feature extraction.
- Comprehensive tests on two renowned retinal image datasets, OCTA-500 and ROSSA, show the robustness of our proposed method. The model’s lightweight design and segmentation speed demonstrates its computational efficiency and potential for broader application across various scenarios.

The paper’s structure is as follows: Section 2 elaborates on the proposed method. Section 3 introduces the experimental data and evaluation metrics and compares the proposed model with others. Section 4 conducts an ablation study to analyze component effects. Section 5 presents the overall conclusion.

## 2. Research Methods

This section outlines the details of our proposed method, referred to as FRNet V2. First, we provide a brief overview of the model framework. Subsequently, we delve into the specifics of each step and block within the framework.

### 2.1. Model Architecture Description

**Figure 2:**
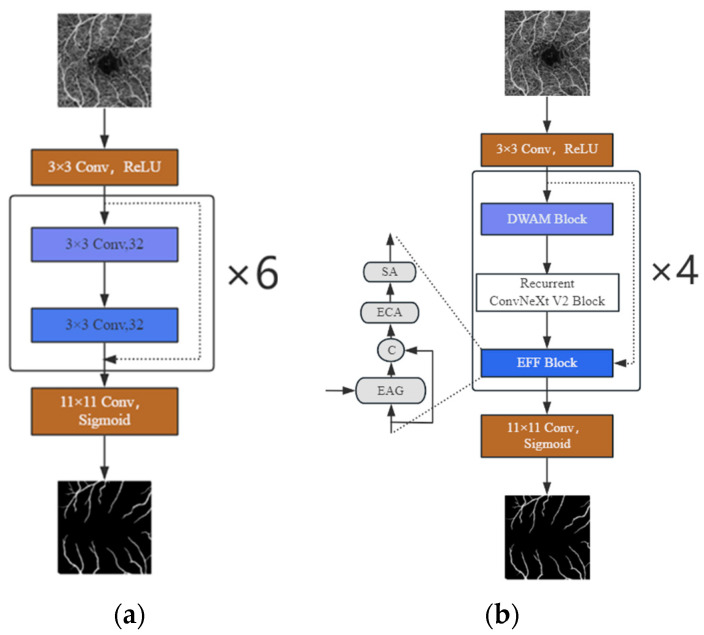
Schematic of the model: (**a**) FRNet-base model [25] and (**b**) FRNet V2 model.

FRNet V2, whose architecture is shown in Figure 2b, consists of ConvNeXt V2 blocks without upsampling or downsampling. It starts with image preprocessing. The preprocessed image undergoes a 3 × 3 convolution (similar to FRNet-base [25] in Figure 2a), then the extracted local features are fed into an improved recursive ConvNeXt V2 block for higher-level and integrated feature extraction. The DWAM attention module and the EFF feature fusion module [26] are combined to enhance features and accuracy, where the EFF feature fusion module includes an enhanced attention gating (EAG) module, a channel attention (ECA) module, and a spatial attention module (SA) module, while the DWAM attention module is explained in Section 2.3 of this chapter. Finally, the segmentation mask is generated via an 11 × 11 fully connected convolutional layer.

The middle part of FRNet V2, similar to FRNet-base, includes four identical convolution operations. It offers the following advantages: First, these operations increase model depth for more feature extraction and better accuracy. Second, without downsampling, it avoids losing small vessel info, achieving high accuracy while reducing parameters.

### 2.2. Improved ConvNeXt V2 Block

**Figure 3:**
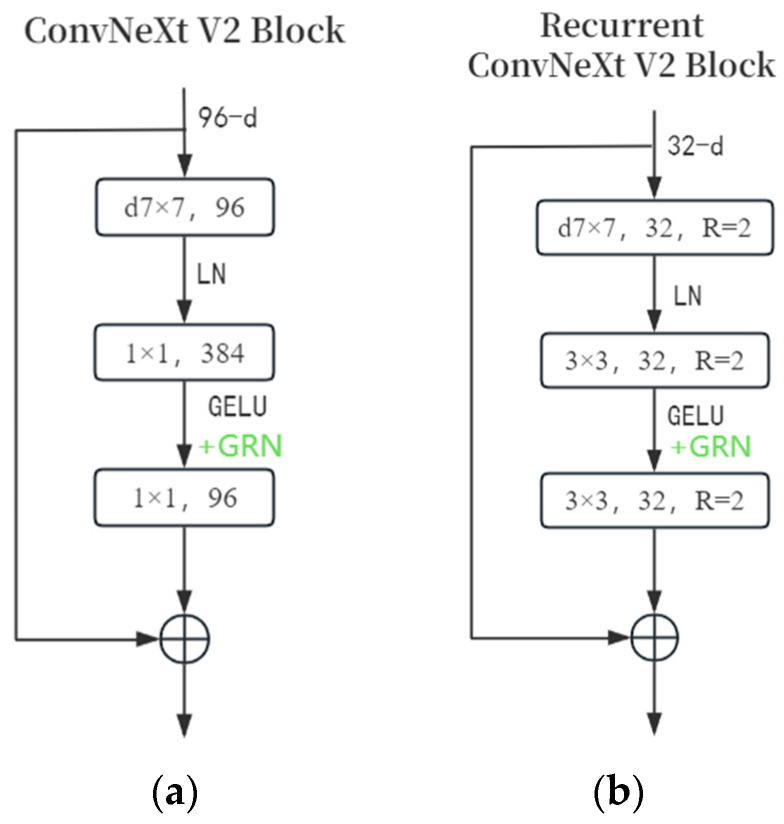
(**a**) Original ConvNeXt V2 block [24] and (**b**) Improved recursive ConvNeXt V2 block.

The original ConvNeXt V2 block (Figure 3a) takes a 96-channel input picture feature. It first applies a 96-channel 7 × 7 depth-separable convolution, followed by data standardization in the LN layer. Then, a 1 × 1 convolution layer boosts the channel number to 384, and the GeLU function activates the data. A GRN layer is added next. The GRN layer normalizes feature maps per channel to enhance inter-channel feature competition. Unlike the BN layer, it can handle any batch size without extra parameters. Additionally, the GRN layer aggregates feature maps, reduces overfitting, and improves model generalization, differentiating it from the ConvNeXt block. Finally, a 1 × 1 convolutional layer restores the channel number to 96. The output is added to the input via residual connection and passed to the next module.

The improvements made to the ConvNeXt V2 block in this paper are presented in Figure 3b. Initially, the convolution channel is set to 32 to expand the receptive field. Next, 1 × 1 pixel convolution is replaced with 3 × 3 convolution, and the concept of recursive convolution is introduced. A recursive mechanism is added to 7 × 7 and 3 × 3 convolutions (R = 2, as proven effective for blood vessel segmentation in [27]). This design offers clear advantages. It significantly reduces the total number of convolution channels, leading to a substantial decrease in the number of parameters. Additionally, it cuts down on computational complexity and the number of convolution operation parameters. This not only enhances the richness of feature representation but also bolsters the model’s expressive and generalization capabilities.

### 2.3. DWAM Attention Mechanism

**Figure 4:**
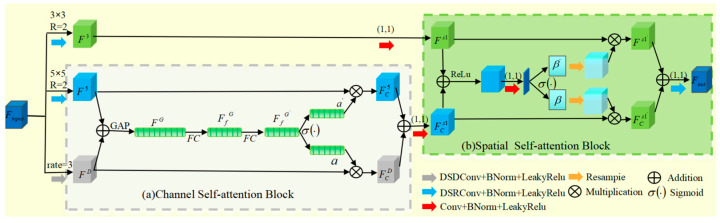
DWAM attention mechanism.

Inspired by the HAAM attention mechanism [28], this paper designs the DWAM attention mechanism. DWAM has three core components: the multi-scale convolutional layer, the channel self-attention block, and the spatial self-attention block. Specifically, the feature map fed into DWAM is processed by three parallel convolutional layers. These layers have different kernel sizes to capture feature information at different scales: a depth-separable recursive 3 × 3 convolutional layer, a depth-separable recursive 5 × 5 convolutional layer, and a depth-separable 3 × 3 expansive convolutional layer (with an expansion rate). Figure 4 shows the DWAM attention mechanism. The feature maps captured by the three convolutional layers can be expressed as:

(1) $$F^{3}=W_{3\times 3}^{DSR}\times F_{input}$$

(2) $$F^{5}=W_{5\times 5}^{DSR}\times F_{input}$$

(3) $$F^{D}=W_{3\times 3}^{DSD}\times F_{input}$$

where $F_{input}\in R^{c\times h\times w}$ represent input feature maps; $W_{3\times 3}^{DSR}$, $W_{5\times 5}^{DSR}$ and $W_{3\times 3}^{DSD}$ represent the matrices of deeply separable recursive convolution 3 × 3, deeply separable recursive convolution 5 × 5, and deeply separable dilated convolution 3 × 3, respectively. $F^{3}\in R^{c\times h\times w}$, $F^{5}\in R^{c\times h\times w}$, and $F^{D}\in R^{c\times h\times w}$ represent feature maps captured by depth separable recursive convolution 3 × 3, depth separable recursive convolution 5 × 5, and depth separable dilated convolution 3 × 3, respectively.

The DWAM attention mechanism mainly includes channel self-attention blocks and spatial self-attention blocks. In the channel self-attention block, the input feature maps are convolved using two depth-separable recursive convolution kernels of different sizes. A 3 × 3 kernel with a dilation rate of 3 and a 5 × 5 kernel generate two sets of feature maps, denoted as $F^{5}$ and $F^{D}$. Each set of feature maps is then separately passed through batch normalization and ReLU activation functions. Subsequently, these two processed sets of feature maps are concatenated and passed through a global average pooling (GAP) layer, which can be expressed as:

(4) $$F^{G}=GAP\left(F^{5}⊕F^{D}\right)$$

where ⊕ represents the addition of elements and $F^{G}$ represents the spliced feature map. Next, the stitched feature maps pass through a fully connected layer, followed by batch normalization and ReLU activation functions to produce new feature maps, as follows:

(5) $$F_{f}^{G}=\sigma_{r}\left(B\left(W_{fc}\times F^{G}\right)\right)$$

where $W_{fc}$, $\sigma_{r}\left(\cdot \right)$, and $B\left(\cdot \right)$ are expressed as a matrix of fully connected layers, a ReLU activation operation, and a batch normalization process, respectively.

Finally, a new feature map is generated through a fully connected layer-${F_{f}}^{G^{\prime }}$, and finally, the feature map is activated by Sigmoid to obtain the channel attention map “a” and “$a^{\prime }$”. These two channel attention maps can help us adaptively extract more representative feature maps from different scales of receptive fields. To achieve automatic feature selection, we use channel attention maps “a” to calibrate the feature maps $F^{D}$ and channel attention maps “$a^{\prime }$” to calibrate the feature maps $F^{5}$. The feature map after channel attention map calibration can be expressed as:

(6) $$F_{C}^{D}=a⊗F^{D}$$

(7) $$F_{C}^{5}=a^{\prime }⊗F^{5}$$

The final feature map $F_{C}^{D}$ and the weighted sum $F_{C}^{5}$ are used as the input of the spatial self-attention block.

The spatial self-attention block first subjects the input feature map to depth-separable recursive 3 × 3 convolution, followed by batch normalization and the ReLU activation function to get a feature map $F^{3}$. Next, 1 × 1 convolution, another batch normalization, and ReLU activation generate spatial features $F^{s1}$.

These spatial features $F^{s1}$ are added to the channel-weighted feature maps $F_{C}^{s1}$ from the channel self-attention blocks. After applying the ReLU activation function to this sum, a new feature map is produced. Then, 1 × 1 convolution, batch normalization, ReLU activation, and Sigmoid activation are carried out on this feature map to generate two spatial attention maps “β” and “$\beta^{\prime }$”.

These two spatial attention maps are resampled to match the number of channels of $F_{C}^{s1}$ and $F^{s1}$. Then, the same operations as in the channel self-attention block are applied to obtain the feature maps $F^{s1^{\prime }}$ and $F_{C}^{s1^{\prime }}$. Finally, a convolution operation generates the final hybrid attention feature map.

The DWAM attention mechanism proposed in this paper has fewer parameters, low computational complexity, and fast running speed. The experimental results show it is highly compatible with our method.

## 3. Experimental Results

### 3.1. Experimental Environment and Hyperparameter Setting

In this study, experiments were run on a Windows 10 system with a 13th Gen Intel i9-13900H 2.60 GHz processor, 32 GB of RAM, and an NVIDIA GeForce RTX 4070 Laptop GPU (24 GB of RAM). The experimental environment was PyCharm, using Python 3.9, CUDA 11.3, and PyTorch 1.11.0. During training, the learning rate was set at 0.0001, the batch size at 32, and the number of epochs at 200. Training would stop if the accuracy did not improve for over 100 consecutive epochs.

### 3.2. Experimental Data

To evaluate the proposed method, this paper performs validation on two common OCTA datasets: the OCTA-500 dataset [29] and the ROSSA dataset [25].

The OCTA-500 dataset contains two subsets of OCTA_6M and OCTA_3M. OCTA_6M contains 300 subjects with a size of 400 × 400, with 180 images for training, 100 images for testing, and 20 images for validation. OCTA_3M contains 200 subjects, with a size of 304 × 304, of which 140 images are used for training, 50 images are used for testing and 10 images are used for validation. The ROSSA dataset contains 918 OCTA images and their corresponding blood vessel annotations, with a size of 320 × 320, in which the Segment Anything Model (SAM) [30] model is used to label the images. The comparison information of the two datasets is shown in Table 1 below.

To augment the data, we employed several techniques: random rotation, the addition of Gaussian noise, random sharpness adjustment, and horizontal, vertical, and diagonal flipping for both datasets. During training and testing, we normalized the images in both datasets.

### 3.3. Evaluation Indicators

In order to evaluate the performance of the model in this paper, the segmentation results are compared with the corresponding labels in this chapter, and the results of each pixel comparison are divided into true positive (*TP*), false positive (*FP*), false negative (*FN*), and true negative (*TN*). Then, accuracy (*Acc*) and Dice coefficient (*Dice*) were used to evaluate the performance of the model. The formulas of the two evaluation indicators in this paper are defined as follows:

(8) $$Acc=\frac{TP+TN}{TP+FN+FP+TN}$$

(9) $$Dice=\frac{2TP}{2TP+FP+FN}$$

In this paper, we use the *Dice loss* as the loss function for our model. The definition of the *Dice loss* function is as follows:

(10) $$DiceLoss=1-\frac{2\left|X\cap Y\right|+\varepsilon }{\left|X\right|+\left|Y\right|+2\varepsilon }$$

where *X* is the predicted image mask, *Y* is the artificially identified real image mask, and *ε* is a small positive number (such as 10^−5^) used to smooth the loss function and ensure its derivability in all cases.

### 3.4. Segmentation Results

**Figure 5:**
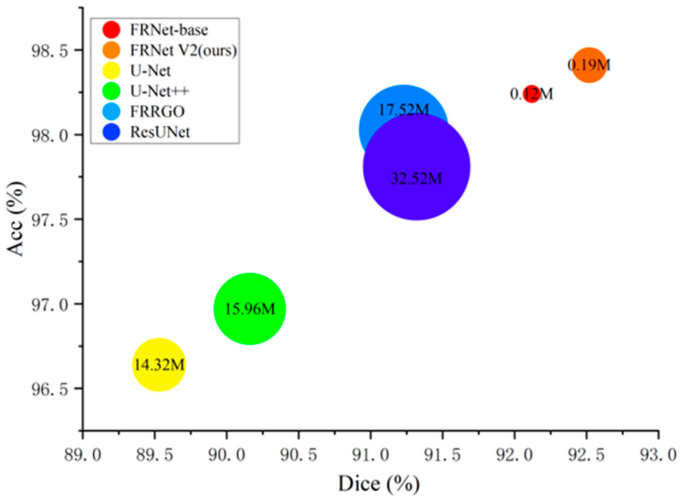
Comparison between different models on the ROSSA dataset.

We conducted vessel segmentation experiments on some of the most popular networks, including U-Net [6], UNet++ [7], ResUNet [10], FARGO [17], and FRNet-base [25]. The segmentation results are presented in Figure 5 and Table 2 (the arrows in the table represent the performance of this model in the evaluation criteria). As can be seen from Table 2, our proposed method attains higher Dice and Acc scores on all datasets. Although the number of parameters and inference time of our method are slightly higher than those of FRNet-base, this is because our model adds an additional convolution operation to the existing convolution operations and incorporates the DWAM attention module and EFF feature extraction module to enhance the feature extraction ability. Despite this, the accuracy achieved by our method is higher than that of FRNet-base, and the model remains stable during training.

From Figure 5, we can see that the number of parameters of FRNet V2 is more than two orders of magnitude lower than other models (e.g., 0.29 M for FRNet V2 vs. 17.52 M for Fargo), and they reason faster than their opponents. This demonstrates that our proposed methods are more efficient, and we expect that they will be more suitable for industrial applications.

**Figure 6:**
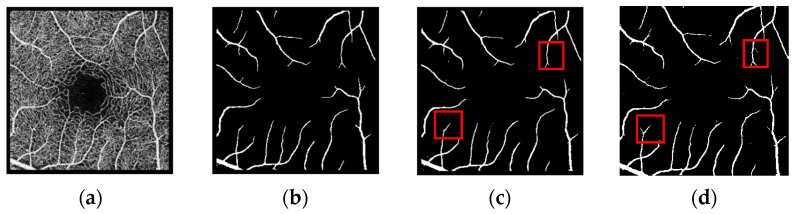
Prediction plot of OCTA-3M dataset. The schematic diagram is as follows: (**a**) OCTA image; (**b**) real label; (**c**) prediction map segmented by the U-Net model; (**d**) prediction map segmented by the model proposed in this paper.

**Figure 7:**
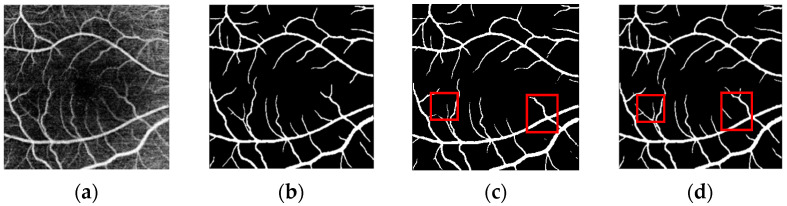
Prediction plot of ROSSA dataset. The schematic diagram is as follows: (**a**) OCTA image; (**b**) real label; (**c**) prediction map segmented by the U-Net model; (**d**) prediction map segmented by the model in this paper.

**Figure 8:**
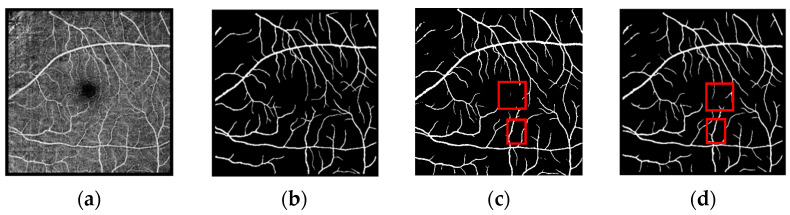
Prediction plot of OCTA-6M dataset. The schematic diagram is as follows: (**a**) OCTA image; (**b**) real label; (**c**) prediction map segmented by the U-Net model; (**d**) prediction map segmented by the model in this paper.

The segmentation prediction plots for the three datasets are shown in Figure 6, Figure 7 and Figure 8. As can be seen in the figures(the red box represents the comparison of different models for the same blood vessel segmentation), our model outperforms the U-Net model, especially in the segmentation of small blood vessels, and the blood vessel connection is complete.

## 4. Ablation Experiment

In this section, we perform ablation experiments to evaluate the effectiveness of the components in FRNet V2, as shown in Figure 3. FRNet V2 can be seen as a network composed of full-resolution convolutions. To verify the impact of each component, we conduct ablation studies using the ROSSA dataset as an example. The results are summarized in Table 3.

- Row 1: Uses only the same modules as FRNet-base.
- Row 2: Replaces the preceding module with the ConvNeXt V2 block. Parameter reduction is due to its deep separable convolution.
- Row 3: Replaces 1 × 1 convolution in the ConvNeXt V2 block with 3 × 3 convolution. Accuracy increases as parameters increase.
- Row 4: Applies recursive convolution, achieving the best accuracy. It does not increase parameters but increases inference time.

We also carried out experiments on the DWAM attention module. To compare it with other attention mechanisms, this paper selected the HAAM attention mechanism and the CBAM [31] attention mechanism. The results are presented in Table 4. The first row of the table indicates the case where no attention module is added. The second row shows the result when the HAAM attention module is added, the third row represents the situation with the CBAM attention mechanism added, and the fourth row corresponds to the addition of the DWAM attention mechanism. From the results in the table, it can be observed that the DWAM attention mechanism proposed in this paper achieves the highest accuracy. Although the number of parameters and inference time of the DWAM attention mechanism are slightly lower than those of the CBAM attention mechanism, the segmentation accuracy of the DWAM attention mechanism is much higher than that of the CBAM attention mechanism.

This paper also conducts experiments on the components of the DWAM attention mechanism itself, and the results are shown in Table 5. The first row indicates the base case with no attention module added. The second row shows the effect after adding the HAAM attention module. The third row represents the situation where ordinary convolution is replaced with deep-separable convolution. We can observe that after adding the HAAM attention module, the accuracy improves, but the number of parameters increases significantly, and the inference time also goes up. When the convolution is replaced, it can be seen that both the number of parameters and the inference time are reduced, while the accuracy is still enhanced. The fourth row represents the combination with recursive convolution, which is equivalent to adding the complete DWAM attention mechanism. Similar to the previous cases, it achieves the best accuracy without increasing the number of parameters, though the inference time increases.

From the experimental results in the above-mentioned tables, it can be seen that the DWAM attention mechanism proposed in this paper is superior to the HAAM in terms of parameter quantity and feature extraction ability, and outperforms the CBAM in segmentation accuracy. This demonstrates its high degree of compatibility with the method proposed in this paper.

As shown in Figure 2a, the FRNet V2 model’s main body has four identically structured convolutional layers. To study the effect of layer number on segmentation, we conducted ablation experiments on the main body. Table 6 shows the results. The data indicate that as the layer number increases, the model’s accuracy, parameter quantity, and segmentation time also increase. However, at five layers, the segmentation accuracy drops, while parameter number and segmentation time continue to rise. At six layers, although accuracy improves, the difference is not significant compared to the four layers. Moreover, the large increase in parameters and the slowdown in segmentation speed compared to the four-layer model go against our method’s goals. Therefore, to strike the best balance, our model uses four identical convolutional layers.

## 5. Conclusions

In this study, we present FRNet V2, a lightweight and efficient neural network for OCTA retinal vessel segmentation. With innovative designs like the ConvNeXt V2 block, deep separable convolution, recursive mechanism, and DWAM attention module, FRNet V2 reduces the model’s burden and speeds up processing while maintaining high accuracy. The experimental results show that FRNet V2 has far fewer parameters compared to other models: it is about 75 times lighter than U-Net, 84 times lighter than U-Net + +, and 171 times lighter than ResUNet. This enables it to work efficiently in resource-constrained environments, such as remote medical facilities. In terms of inference speed, FRNet V2 is about 1.3 times faster than U-Net + + and nearly two times faster than ResUNet, which is crucial for rapid diagnosis. Regarding accuracy, on OCTA_6M, OCTA_3M, and ROSSA datasets, FRNet V2 outperforms others in Dice coefficient and Acc accuracy, achieving Dice values of 89.10%, 91.63%, and 92.52%, and Acc values of 98.20%, 98.97%, and 98.41%, respectively, demonstrating its adaptability and robustness.

In conclusion, FRNet V2 surpasses existing models in parameter quantity, inference speed, and segmentation accuracy. As a lightweight and efficient solution, it has significant advantages in practical applications, especially in real-time analysis with limited computational resources. However, the model proposed in this paper still needs to be optimized. For instance, in the selection of activation functions, many excellent activation functions have been proposed successively, such as ASU [32] and SwishReLU [33]. In the future, we plan to further optimize the model and explore its potential in other medical imaging tasks.

## Figures and Tables

**Table 1:** OCTA-500 and ROSSA dataset specific information.

Dataset	OCTA-500	ROSSA
Number of images	500	918
Subdatasets	OCTA_6M and OCTA_3M	Train, Test, and Val
Num of train/test/val	320/150/30	718/100/100
Size	400 × 400 (OCTA_6M) 304 × 304 (OCTA_3M)	320 × 320

**Table 2:** Comparison between different methods.

Method	Dice (↑)	Acc (↑)	Param (↓)	Time (↓)
OCTA_6M				
U-Net	85.03	95.21	14.32 M	20.2 ms
U-Net + +	85.67	95.73	15.96 M	25.7 ms
ResUNet	88.10	96.03	32.52 M	32.4 ms
FARGO	89.01	98.12	17.52 M	29.6 ms
FRNet-base	88.85	98.02	0.12 M	15.3 ms
FRNet V2	89.10	98.20	0.19 M	21.8 ms
OCTA_3M				
U-Net	88.35	95.45	14.32 M	17.4 ms
U-Net + +	88.64	95.98	15.96 M	21.2 ms
ResUNet	90.03	96.18	32.52 M	26.3 ms
FRRGO	91.21	98.12	17.52 M	24.5 ms
FRNet-base	91.15	98.84	0.12 M	12.1 ms
FRNet V2	91.63	98.97	0.19 M	13.5 ms
ROSSA				
U-Net	89.53	96.64	14.32 M	18.9 ms
U-Net + +	90.16	96.97	15.96 M	23.9 ms
ResUNet	91.32	97.81	32.52 M	28.7 ms
FRRGO	91.23	98.03	17.52 M	27.5 ms
FRNet-base	92.12	98.24	0.12 M	13.8 ms
FRNet V2	92.52	98.41	0.19 M	14.9 ms

**Table 3:** Ablation experiments of ConvNeXt V2 module in FRNet V2.

Component	Dice	ACC	Param	Time
Residual Block	91.89	98.28	0.11 M	9.1 ms
ConvNeXt V2 Block	91.23	97.91	0.07 M	7.5 ms
1 × 1⇒3 × 3	91.97	98.36	0.12 M	10.5 ms
+Recurrent	92.27	98.38	0.12 M	11.6 ms

**Table 4:** Comparative experiments of DWAM attention mechanism in FRNet V2.

Component	Dice	ACC	Param	Time
Without Attention	92.27	98.38	0.12 M	11.6 ms
With HAAM	92.39	98.39	0.59 M	18.4 ms
With CBAM	92.29	98.38	0.18 M	13.2 ms
With DWAM	92.52	98.41	0.19 M	14.9 ms

**Table 5:** Ablation experiments of DWAM module in FRNet V2.

Component	Dice	ACC	Param	Time
Without Attention	92.27	98.38	0.12 M	11.6 ms
With HAAM	92.39	98.39	0.59 M	18.4 ms
Conv⇒DSConv	92.45	98.40	0.19 M	11.9 ms
+Recurrent	92.52	98.41	0.19 M	14.9 ms

**Table 6:** Ablation experiments with different number of layers in the model.

Model Layers	Dice	ACC	Param	Time
Layer two	92.27	98.34	0.09 M	7.2 ms
Layer three	92.33	98.38	0.14 M	11.1 ms
Layer four	92.52	98.41	0.19 M	14.9 ms
Layer five	92.37	98.40	0.24 M	19.0 ms
Layer six	92.54	98.42	0.29 M	24.5 ms

## Data Availability

The OCTA-500 Dataset: IPN-V2 and OCTA-500: Methodology and Dataset for Retinal Image Segmentation; The ROSSA Dataset: https://github.com/nhjydywd/OCTA-FRNet (accessed on 2 March 2025). The implementation of FRNet V2 is available at https://github.com/wangliang612/OCTA-FRNet--V2 (accessed on 2 March 2025).
